# Development of a Reliable High-Performance WLP for a SAW Device

**DOI:** 10.3390/s22155760

**Published:** 2022-08-02

**Authors:** Zuohuan Chen, Daquan Yu

**Affiliations:** School of Electronic Science and Engineering, Xiamen University, Xiamen 361005, China; xmuchenzh@stu.xmu.edu.cn

**Keywords:** surface acoustic wave filter, wafer-level packaging, laser drilling, finite element model, reliability

## Abstract

In this paper, we present wafer-level packaging technology for surface acoustic wave (SAW) filters with higher long-term reliability and better electrical performance. This article focuses on the package structure, fabrication processes, and reliability for the SAW filter wafer-level package (WLP). The key processes, including cavity wall (CW) dam formation through non-photosensitive film vias development using a laser drilling process, a redistribution layer (RDL), and ball-grid array formation are developed. In addition, a numerical study based on the finite element model has been conducted to analyze the stress distribution of Cu RDL traces. In addition, the CW dam and the roof layer are covered with polymer, which solves the delamination problem between the CW dam and the substrate. Meanwhile, after practical verification, the SAW filter WLP was resistant to encapsulating pressure using a high elastic modulus capping material, which solved the collapse problem. Additionally, a comparison of the RF filter package’s electrical performance following the preconditional level 3 and unbiased highly accelerated stress test revealed no differences in insertion attenuation across the passband (<0.2 dB, standard value: 1 dB). The final packages passed the reliability tests in the field of consumer electronics.

## 1. Introduction

The recent advent of 5G networks has necessitated the assistance of acoustic wave device volume creation. Acoustic wave devices, such as surface acoustic wave (SAW) and bulk acoustic wave (BAW), are primarily utilized in the front ends of current mobile transceiver systems to filter out desirable frequencies. Many specialists have worked to reduce the size or number of SAW/BAW devices or to eliminate them entirely through design or packaging changes [1,2,3]. However, there are currently no competitive devices that offer the same performance at the same size and price. According to Qorvo’s estimates, the market for radio frequency (RF) filters in mobile phones is worth USD 12.83 billion. This unlikely to decrease. This paper aims to capture the rationales behind these approaches to new packaging approaches, such as novel topologies, new materials, and enhanced simulation modeling achieving a higher performance and lower cost of SAW devices [4,5,6]. A series of evaluations and investigations into the development of high-performance wafer-level packaging (WLP) for SAW devices are offered in order to achieve this goal.

### 1.1. Historical View of SAW Device Package

SAW device packaging began with a hefty ceramic container that was unsuitable for mobile radios. SAW packages were primarily wire-bonded ceramic, metal, and plastic containers until 2001. Then, EPCOS introduced the chip-sized SAW package (CSSP) [2,3]. Flip-chip technology was employed to replace wire bonding in this package form. The flip-chip technique connects the chips to a high-temperature confirmed multilayer ceramic (HTCC) substrate, which is then coated by polymer lamination, which is a characteristic packaging method utilized only in low-frequency devices [2,3].

WLP is the next step in miniaturization and cost reduction [7,8,9,10,11,12,13,14]. In WLP, one or two organic or inorganic layers on top of the piezoelectric crystal, such as a lithium (LiTaO_3_, LT) piezoelectric substrate, form cavities above the active portions of the chip, which can protect the susceptible SAW device structure from probable injuries, such as dust or corrosion. Different processes, such as the production of polymer caps utilizing a sacrificial layer or dry film resist lamination techniques, are used to create these hollow structures [9,10,11]. The SAW devices are also sealed with a stiff cap structure using wafer-to-wafer or chip-to-wafer bonding technologies. The cap layer made of glass or polymer film with via interconnects were formed to contact the external circuits and the chip’s pads [15,16]. Partially limiting the interdigital transducer (IDT) regions while leaving the SAW devices’ IOs available is more cost-effective. A self-packaged SAW device using a boundary acoustic wave, which does not require cavities or a particular processing approach for making sturdy packages, is even better from a functional standpoint. However, because it uses Au interdigital transducers deposited on LiNbO_3_ substrates, which has a greater cost, this filter is not advantageous in terms of cost and filter qualities. Typically, these devices operate in the mobile environment at frequencies ranging from 1040 to 1120 MHz, which do not match the high-frequency requirement.

Because the range of existing technologies is so broad, there is no perfect solution in terms of key evaluating indicators such as low cost, process risk, reliability performance, degree of miniaturization, and compatibility with various microelectromechanical systems (MEMS) and complementary metal oxide semiconductor (CMOS) technologies on a wafer [17,18]. We aimed to create a reliable high-performance WLP for SAW device processing flow in this study, with a particular focus on keeping the low cost and post-wafer-level process flow as short as possible to reduce the chance of process failure. The process of developing a dependable high-performance SAW device WLP is described in the following paragraphs. We employed devices developed for the B41 band application with operation frequencies ranging from 2.496 GHz to 2.690 GHz as SAW filter test devices. SAC solder balls were used in the final SAW device packaging, which was mounted to external circuitry, like a circuit board; it was flipped over, so that its top side faced down and its pads were aligned with matching pads on the external circuit; then, the solder was reflowed to complete the interconnect. In Section 1, we present an overview of packaging technologies and propose various SAW filter WLP configurations to fit various application scenarios. Section 2 describes the WLP process flow and common failure mechanisms, which include interfacial delamination and redistribution layer (RDL) fracture. Section 3 builds a wafer-level and a single-die simplified finite element (FE) model for von Mises stress and collapse prediction. Both FE models were shown to be accurate through theoretical calculations and actual experimental results’ comparison. We presented an in-depth look at the creation and reliability enhancement of the wafer-level molded package based on the proposed packaging models for the diverse structural design of the SAW device. The packages were tested for reliability, as detailed in Section 4, and all of them passed. Finally, in Section 5, some observations are made.

### 1.2. SAW Device Package Design

**Figure 1:**
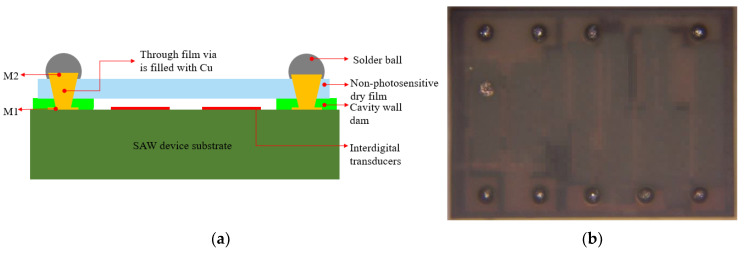
(**a**) The schematic of the cross-sectional SAW filter WLP structure; (**b**) the 3D WLP SAW filter’s top view following BGA creation.

In this paper, the SAW filter with a size of 1.58 mm × 1.18 mm was packaged. There were eight pads on the front side, and each pad had a size of 70 µm× 70 µm. The WLP structure of the SAW device is presented in Figure 1.

As shown in Figure 1, the SAW filter package consisted of a SAW filter substrate with IDTs and pads, a CW dam (also called a wall layer), non-photosensitive dry film capping (also called a roof layer), an RDL, and solder ball. There were three metal layers in the package; the first layer was metal 1 (M1), the second layer was the through film vias (TFV), used to connect the top and bottom metal, and the third layer was M2. The top and bottom diameters of the CW dam and roof layer were 50 and 70 μm (dimensional tolerance: ±3 μm), respectively. The thickness of the CW dam was 15 μm, while the non-photosensitive dry film layer was 20 μm thick. The solder balls were 110 μm in diameter and 70 μm in height, respectively. Meanwhile, cavities with a size of 980 μm × 320 μm in a thin-film capping acoustic package were designed.

## 2. Process Flow of the SAW Device’s WLP and the Typical Failure Mode

This WLP technique is a real chip-scale packaging method that enables packages to maintain the same size as a die. A new efficient WLP technique created a gas-filled cavity structure for SAW filter packages, using non-photosensitive film lamination technology, with high strength and a high elastic modulus [7,8,9,10,11,12,13,14,19].

### 2.1. Process Flow of SAW Device WLP

**Figure 2:**
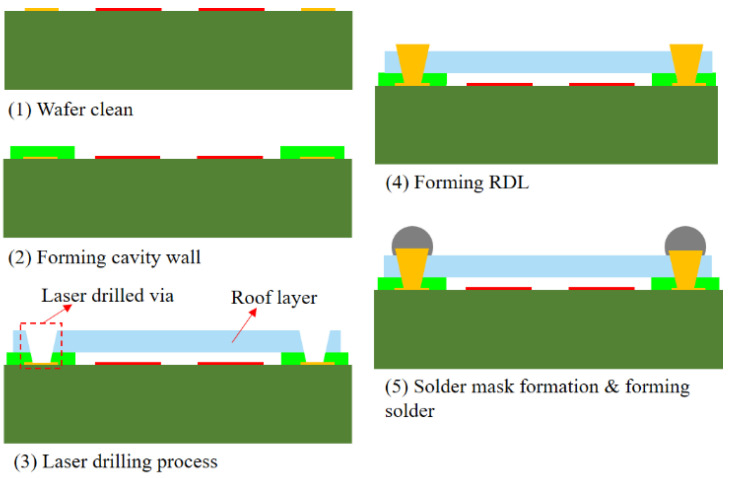
The process flow of the 3D WLP package.

**Figure 3:**
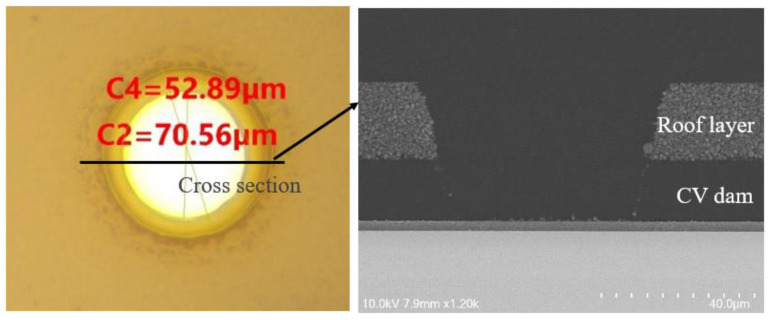
Top view of the opening size of the CW dam and roof layer.

The process flow of the SAW device WLP is depicted in Figure 2, and the details are listed as follows: (1) we prepared a 220 μm SAW filter substrate wafer with IDTs and an M1 layer and used the lithography process to make a CW dam layer. (2) A non-photosensitive dry film was laminated on the CW dam layer using a roller lamination method to make an individual cap for each IDT. As shown in Figure 3, we used laser drilling to drill TFVs with different diameters [20,21]. Meanwhile, no delamination of the roof layer in the top of the CW dam layer was found from scanning electron microscopy (SEM). The non-photosensitive dry film was a non-hermetic material; the inside of the microcavity had the same pressure as the outside of the device after forming the cavity wall. (3) Physical vapor deposition (PVD) and the RDL process was used to fill Cu into TFVs and form the M2 layer. (4) The solder balls were dropped on the M2 layer by stencil printing and a reflow process. The diameter and height of the solder ball were 110 μm and 70 μm, respectively. The wafer was diced into individual packages after the final solder-balling procedure.

### 2.2. The Experimental Results of Typical Failure Mode

**Figure 4:**
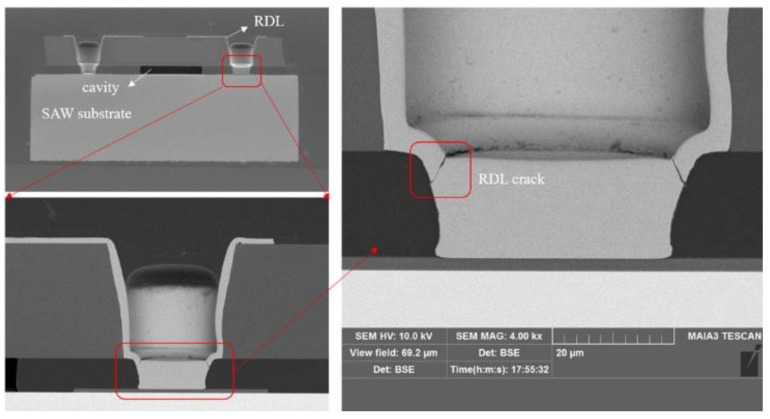
SAW filter package failure mode. (This refers to an RDL crack).

**Figure 5:**
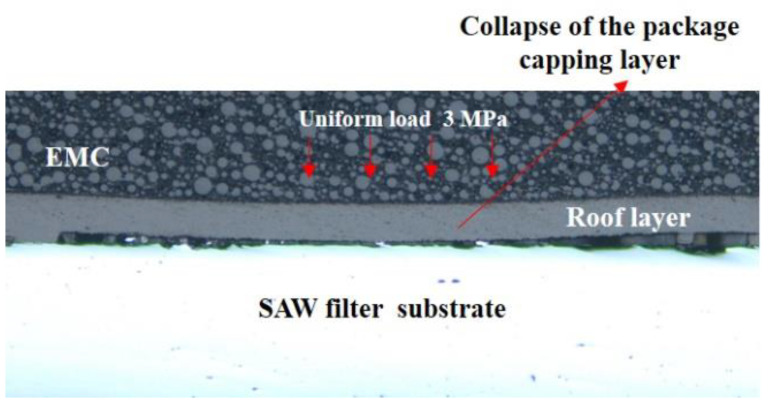
Collapse of the SAW WLP package.

The reliability of the SAW filter package is a major concern for the RF front-end passive devices. Many types of failures may happen during assembly, qualification tests, and operation. Typical failure modes are shown in Figure 4 and Figure 5, such as an RDL crack in the TC test and capping layer collapse of the package. Meanwhile, the RDL structure may affect the thermomechanical performance of the solder joints or SAW devices, in severe cases, thus causing the entire device structure to be destroyed. All of these failures will cause pollution or damage to the IDTs.

In general, a mismatch in the coefficient of thermal expansion (CTE) between different materials in a WLCSP structure can induce high thermal stress. Because the performance of the package may be impacted by the design structure and material characteristics, such as the thickness of the vias sidewall Cu plating and material properties of both the CW dam and roof layer, it is necessary to investigate the impact of these aspects.

To ensure reliability, for a SAW filter WLP, it was necessary for us to investigate the causes of typical failure models and optimize the package structure to meet the reliability requirements of consumer electronics.

## 3. FE Model of a SAW Filter’s WLP

### 3.1. Single-Die FE Model to Solve the RDL Crack Failure Mode

**Figure 6:**
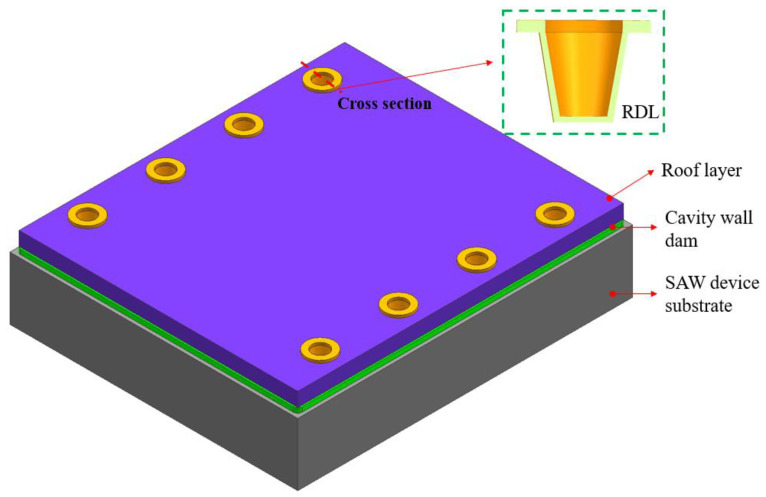
The single-die FE modeling.

To investigate the stress distribution of Cu RDL traces, an FE model was constructed to calculate the maximum primary stress at each phase of the manufacturing process flows, focusing on the different Cu thicknesses of the vias sidewall at the steady-state cooling process (temperature conditions varied from 260 °C to 22 °C) to achieve high dependability [22,23]. The package structure model was developed and simplified into four parts in this model, as illustrated in Figure 6, comprising the RDL, roof layer, CW dam layer, and SAW device substrate (from top to bottom), and the material properties are listed in Table 1.

**Figure 7:**
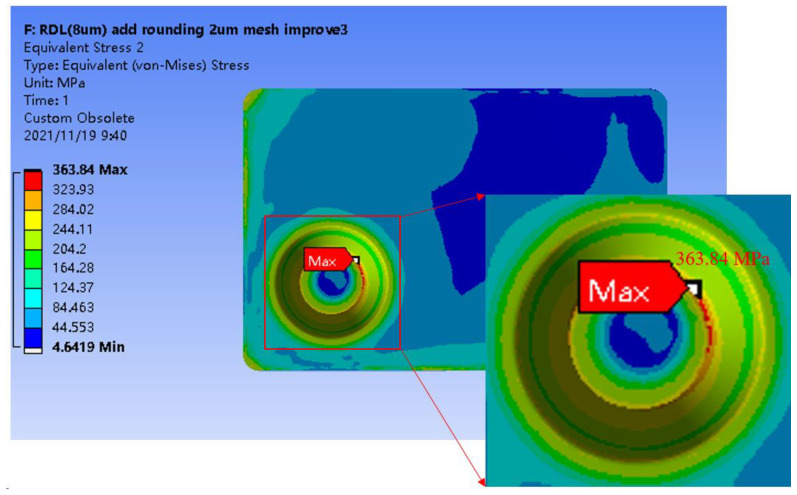
Stress distribution of the thickness of the Cu plating at 8 µm during the cooling-down stage of the curing process.

The Cu thickness of the via sidewall was adjusted to 2 µm, 8 µm, and filled in the simulation setting. Temperature conditions were varied from 260 °C to 22 °C. Then, 260 °C was chosen as the reference temperature. Figure 7 depicts the stress distribution. The max stresses for the via sidewall Cu thickness at 2 µm, 8 µm, and filled were 416.07 MPa, 363.84 MPa, and 350.45 MPa, respectively, which means the stress decreased as the Cu thickness of the via sidewall increased, as presented in Figure 7 and Table 2. In a followup design structure, the full-via Cu electroplating process was performed.

### 3.2. Effect of Geometric Parameters on Stress Distribution

**Figure 8:**
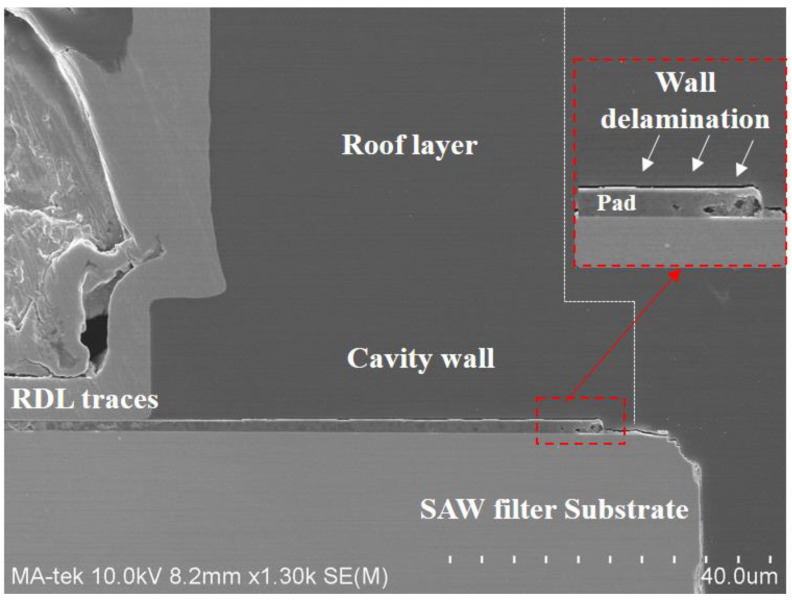
SAW filter package failure modes after TC test. (The arrow, in this Figure, refers to a magnified image of the delamination between the wall layer and substrate).

It is essential to thoroughly research the geometric characteristics and material parameters, since they might have an impact on the dependability of the SAW filter package. In Figure 8, we show the typical wall-layer delamination in the bottom of the pad after the temperature cycling test (TCT). The performance of high-temperature composites can be significantly affected by the presence of residual stresses. These stresses arise during the cooling processes from fabrication to room temperature due to a mismatch in the CTE of the various materials. This will cause wall delamination and an RDL crack.

**Figure 9:**
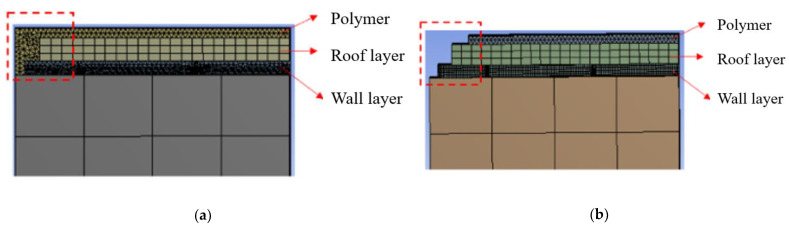
Structural comparison of (**a**) top-side molded WLP with (**b**) typical fan in SAW device package.

A greater comprehension of the adhesion processes will accelerate the development of microelectronic and SAW filters in order to increase the dependability performance. As a typical wall-layer delamination can be influenced by geometric parameters, it is necessary to investigate those factors in depth. Figure 9 shows the cross-sectional structure of the improved SAW filter WLP. The most significant feature is that polymer materials were added to the SAW device to give extra protection to the exposed bard wall and roof layer on the sidewall. In this way, smaller peeling stress between the wall layer and SAW filter substrate was obtained without wall layer delamination.

**Figure 10:**
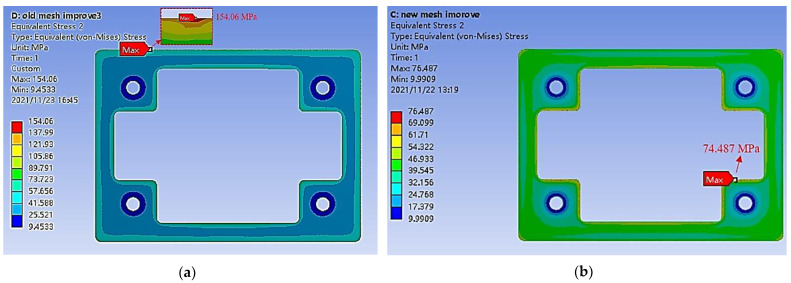
The maximal equivalent stress value distribution comparison of (**a**) the typical fan in the SAW filter structure without a polymer layer mold and with (**b**) the top-side molded SAW filter WLP.

Figure 10 shows the maximal equivalent stress value distribution on the bottom surface of the wall layer. We find that the maximum equivalent stress appears at the bottom edge of the wall layer. Meanwhile, we compared the unimproved and improved SAW filter WLP, and the equivalent (von Mises) stresses were 154.06 MPa and 74.487 MPa, respectively (the maximal stress is marked in Figure 10), which means the maximum stress value decreased by 51.65% on average.

### 3.3. Deflection Prediction of Different Cavity Sizes

In the case of the package capping layer collapse, a non-photosensitive film with high strength and high elastic modulus has become more and more attractive for the wafer-level SAW filter packages because it exhibits excellent pressure capability.

**Figure 11:**
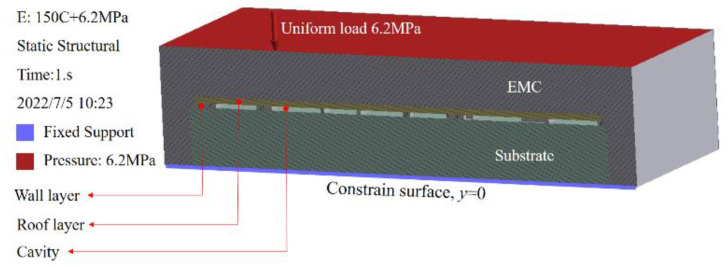
Collapse model and boundary conditions.

We developed a deflection prediction model to simulate the collapse of the package capping layer in order to forecast the cavity deflection under pressure [5,24]. A single SAW filter WLP collapse model and its boundary conditions are shown in Figure 11. The restrained surface was stationary (it means that y=0). After the cavity was created, epoxy molding compound (EMC) was applied to the whole device, and then a consistent load was placed on top of the EMC.

**Figure 12:**
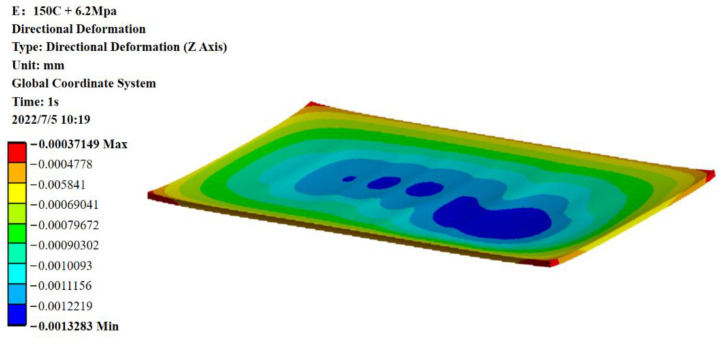
The collapse result after the roof layer process obtained by applying 6.2 MPa pressure to the FE model.

**Figure 13:**
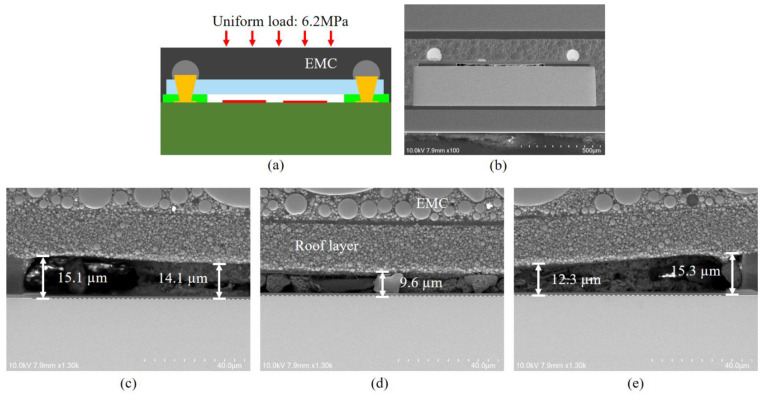
Experimental results of collapse test by applying 6.2 MPa pressure. (**a**) Schematic of molding pressure test; (**b**) indicates the SEM image of the SAW device molding pressure test; (**c**–**e**) indicate the degree of collapse of the cavity at the left, middle and right positions, respectively.

Through discussion of the WLP structure of the SAW filter, the maximum collapse of the cavity with a size of 980 µm × 320 µm in the device was simulated. This was performed by studying the collapse calculation of the device using the finite element simulation model. Following simulation validation, Figure 12 shows that the quantity of collapse at a higher mold pressure of 6.2 MPa was approximately 1.32 μm. After practical verification, the highest amount of collapse in the center of the cavity was estimated to be around 5.4 μm at the mold pressure of 6.2 MPa, as shown in Figure 13. At this point, the cavity’s height was 9.6 μm.

The difference between the measured and simulated results was small. In the actual test of the cavity collapse resisting molding pressure, there were also factors such as the error of measurement (tolerance range: ±2 μm) and substrate warpage, which could not be simulated in the simulation software, and the simulation results were only used as a directional guide. The collapse issue brought on by the SAW filter WLP’s encapsulation pressure was resolved by using a non-photosensitive dry film with a high modulus of elasticity as a capping layer, which also lowered the failure risk of devices and modules.

## 4. Reliability Evaluation

SAW filters and BAW filters are examples of electronic and RF equipment that are used in a variety of mechanically and thermally demanding environments. We carried out typical reliability testing to certify the SAW filter’s WLP, as shown in Table 2. Before reliability testing, preconditioning was conducted to simulate the effects of the board assembly. The samples went through a 192-h soak procedure at 30 °C and 60% relative humidity, as well as a 24-h baking process at 125 °C. The samples were then reflowed three times by a temperature profile with a 260 °C peak. The unbiased highly accelerated stress test (uHAST) temperature storage test at 130 °C for 96 h was carried out for reliability evaluation with the goal of evaluating dependability. The Joint Electron Device Engineering Council (JEDEC) standards were referenced in the test circumstances.

**Figure 14:**
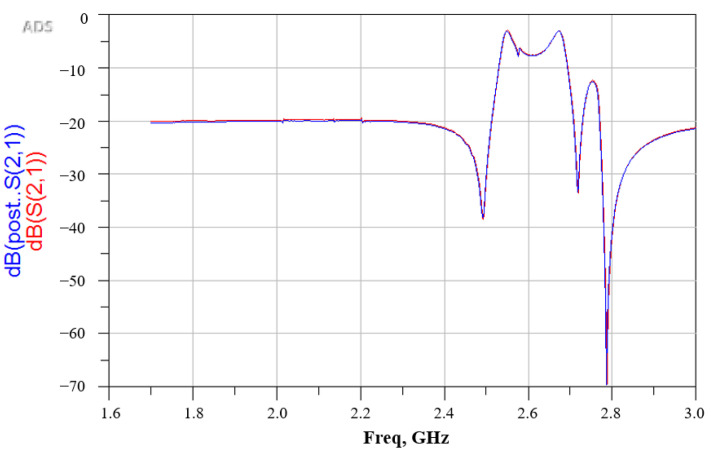
Measurement results of frequency response before and after reliability test.

**Figure 15:**
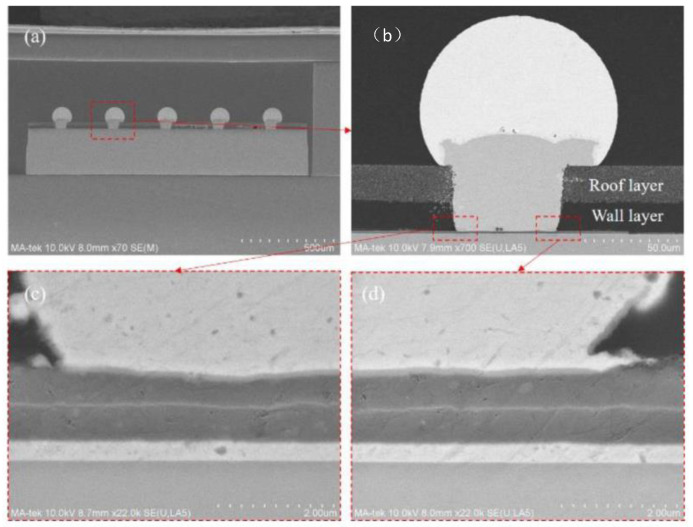
SEM image of the RDL of a single package after uHAST 96 h. (**a**) Cross-sectional picture of the SAW Device WLP with solder ball. (**b**) Interconnection structure of the chip’s pad and the solder ball by TFV. (**c**,**d**) indicate the interconnection structure on the left and right side of the chip’s pad, respectively.

Each reliability test used 30 samples. In Table 3, the dependability findings are summarized. There was no variation in the insertion attenuation across the passband (<0.2 dB) for the SAW filter package following the uHAST test, as shown in Figure 14 [3]. The chosen samples were characterized and cross-sectioned by SEM after the reliability test and the subsequent electrical testing, as shown in Figure 15. No delamination was found between the wall layer and the SAW filter substrate after uHAST 96-h. There was no crack at the interface between the bottom of the via and the Al pad, and the RDL profile was normal, as it had been before the reliability test.

## 5. Conclusions

In this paper, a construction procedure for a 3D WLP for a SAW filter using non-photosensitive dry film lamination and laser drilling technologies was presented. Due to its effective packaging process, it can provide a low-cost alternative to a conventional packaging method for smaller SAW filter applications. The main conclusions are described as follows.

(1) A wafer-level lamination process was conducted on the SAW filter substrate with the CW dam layer together before the electrical interconnection process. Laser drilling was used to form the connection vias, which was characterized by a gradual increase in diameter from the bottom to the top of the TFVs, and the side walls were smooth without steps.
(2) Parameters, such as material properties and geometry, affect the maximum stress of the package significantly. Following practical verification, the cavity collapse amount was projected to be about 6.6 μm at a larger size of 980 μm × 320 μm, resolving the collapse problem caused by the encapsulating pressure of the SAW filter WLP. In terms of the effect of the geometric parameters on stress distribution, by comparing the top-side molded with the conventional SAW filter WLP, the maximum von Mises values were 74.487 MPa and 154.06 MPa, respectively, with a 51.65% reduction.
(3) After Pre Con L3 and uHAST 96-h, no electrical breakdown was seen during the reliability tests. The results show that the proposed SAW filter package based on 3D WLP technology is reliable for large-scale industrial manufacture. We infer from this that our technique can significantly enhance the development and use of consumer market band applications.

## 6. Patents

Zuohuan Chen, Daquan Yu, Jiang Feng, et al. A Wafer-level packaging structure and method for filter. CN Patent 202210528805.8. 16 May 2022.

## Figures and Tables

**Table 1:** Material properties of the two single-die models.

Items	Young Modulus	Poisson Ratio	CTE
SAW device substrate	230 Gpa	0.22	16.1 ppm/°C 4.1 ppm/°C
Cavity wall layer	4.4 Gpa	0.34	65 ppm/°C
Roof layer	9 Gpa	0.34	CTE1: 18 ppm/°C (25–150 °C) CTE2: 45 ppm/°C (150–240 °C)
Cu	119 Gpa	0.326	17.5 ppm/°C

**Table 2:** Statistics of the stress value during the different thicknesses of Cu plating.

Cu Thickness	Stress Point (MPa)	Ratio
2 µm	416.07	1.00
8 µm	363.84	1.14
filled via	350.45	1.19

**Table 3:** Items and results of reliability test.

Items	Conditions		Failure Rate (%)	Result
Pre-Con L3	Bake	125 °C/24 H	0.00	Pass
	Soak	30 °C/60%/192 H	0.00	Pass
	Reflow	260 °C (+5/−0) 3×	0.00	Pass
uHAST	130 °C/85% RH, 96 H		0.00	Pass

## Data Availability

Not applicable.
